# Supplementary material for: Fragmenting networks by targeting collective influencers at a mesoscopic level
Source: Sci Rep. 2016 Nov 25;6:37778. doi: 10.1038/srep37778 (PMC5122919; doi:10.1038/srep37778)
Supplement: Supplementary Information [file srep37778-s1.pdf]

Supplementary Information: Fragmenting networks by targeting collective influencers at a mesoscopic level

Teruyoshi Kobayashi and Naoki Masuda

S1. CBCI ALGORITHM UNDER AN ONLINE UPDATING OF COMMUNITY STRUCTURE

As described in the main text, the CbCI algorithm carries out community detection only once at the beginning of the node removal process. This saves computation time but may worsen the performance of the immunization because the organization of communities may change as we remove nodes. In this section, we investigate the impact of recalculating the community structure repeatedly during the node removal process. We recalculate the partitioning of the network every time we remove $10^{-3}N$ nodes. By feeding the most up-to-date partitioning to the CbCI algorithm, we determine the tentative order of the node removal. Then, we reinsert the nodes in the same manner as the original CbCI algorithm does. The entire reinsertion procedure uses the community structure of the original network, i.e., that determined before the node removal. We reinsert the nodes one by one. We focus on Infomap and Walktrap, with which the CbCI algorithm performs the best. We use AS-1, PGP, and CA-GrQc networks for illustration.

The immunization results for the CbCI algorithm with online updating of the community structure are compared with those for the original CbCI algorithm and the CI algorithm in Fig. S1. The figure indicates that online updating of the community structure improves the performance of the CbCI algorithm in some cases but not in others.

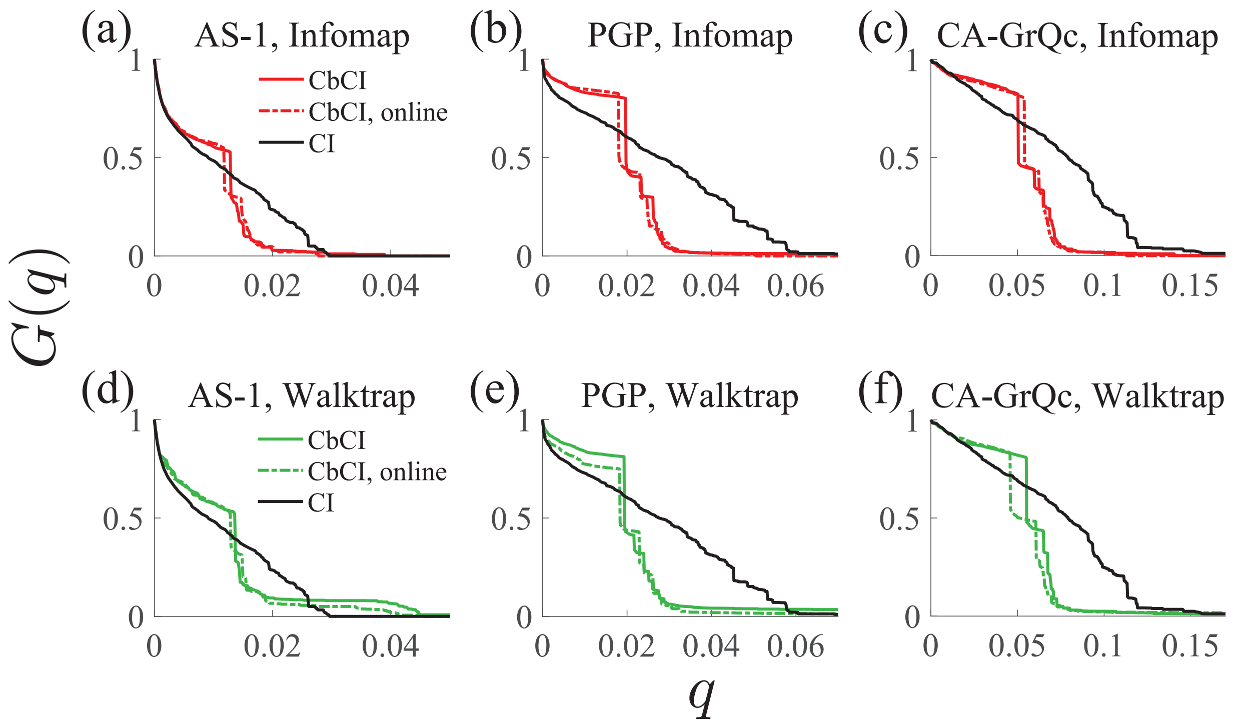

FIG. S1. Online updating of the community structure in the CbCI algorithm. In each panel, the size of the LCC under the original CbCI algorithm, the CbCI algorithm with online updating of the community structure, and the CI algorithm is plotted against q.

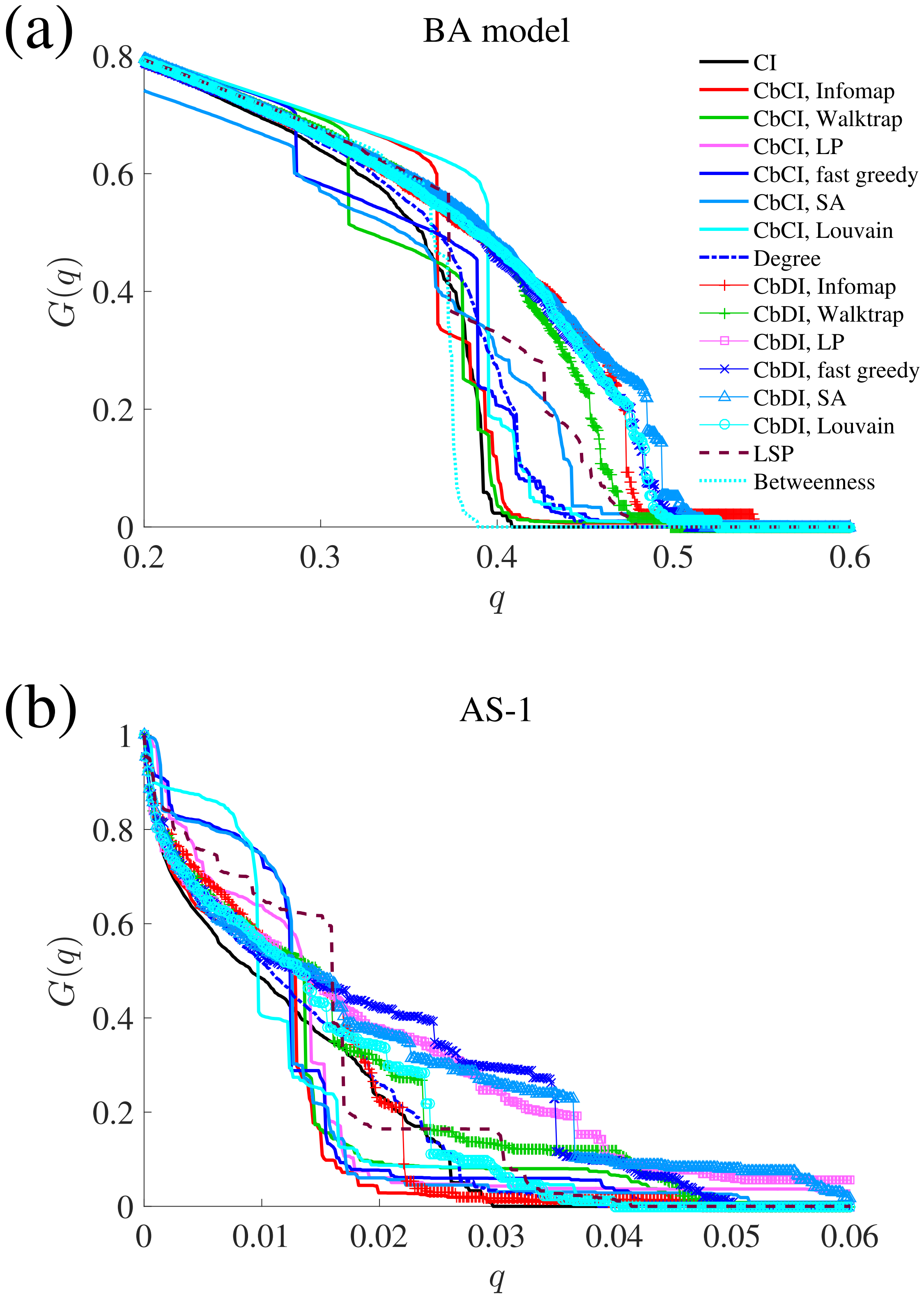

FIG. S2. Normalized size of the LCC, $G(q)$, plotted against the fraction of removed nodes, q, for different networks and immunization algorithms. In (a), the results for the label-propagation community detection algorithm are absent because it yields no community (i.e., $N_c = 0$) for this network.

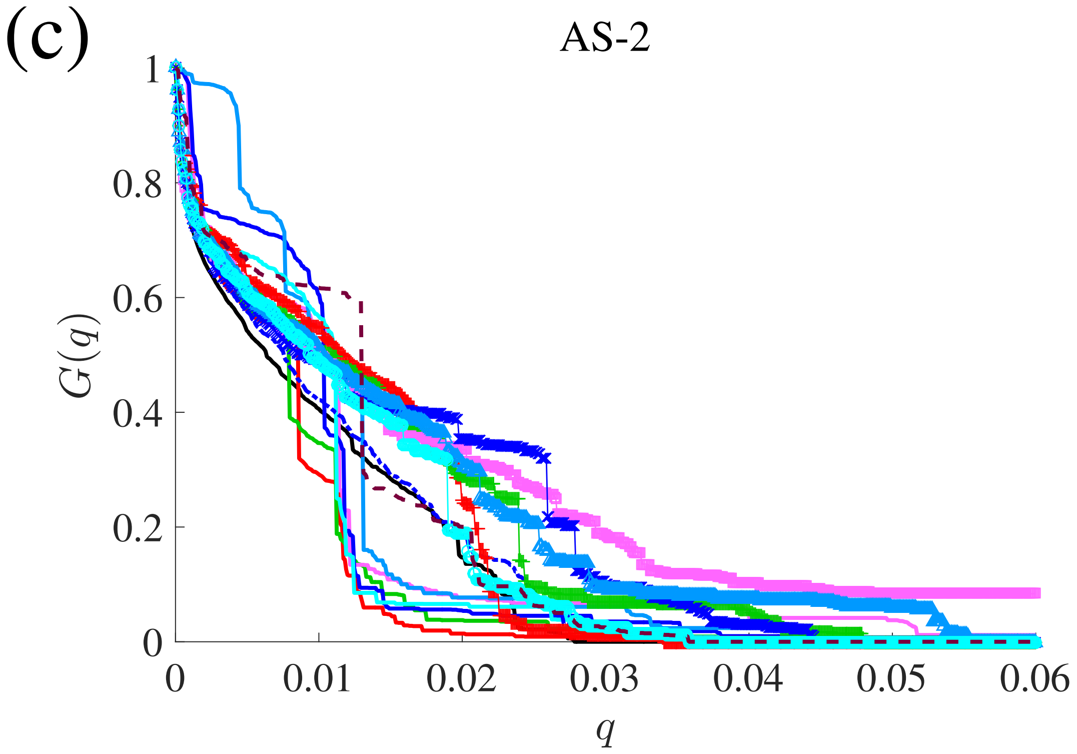

(c)
AS-2
1
0.8
0.6
0.4
0.2
0
G(q)
0
0.01
0.02
0.03
0.04
0.05
0.06
q

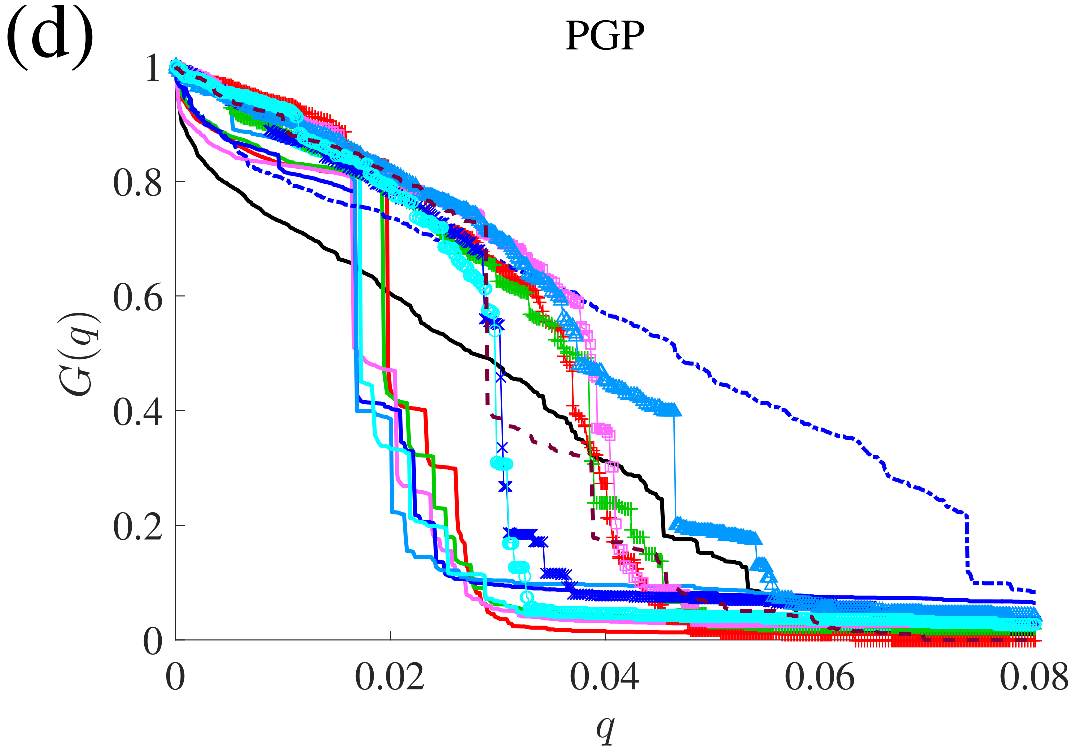

(d)
PGP
1
0.8
0.6
0.4
0.2
0
G(q)
0
0.02
0.04
0.06
0.08
q

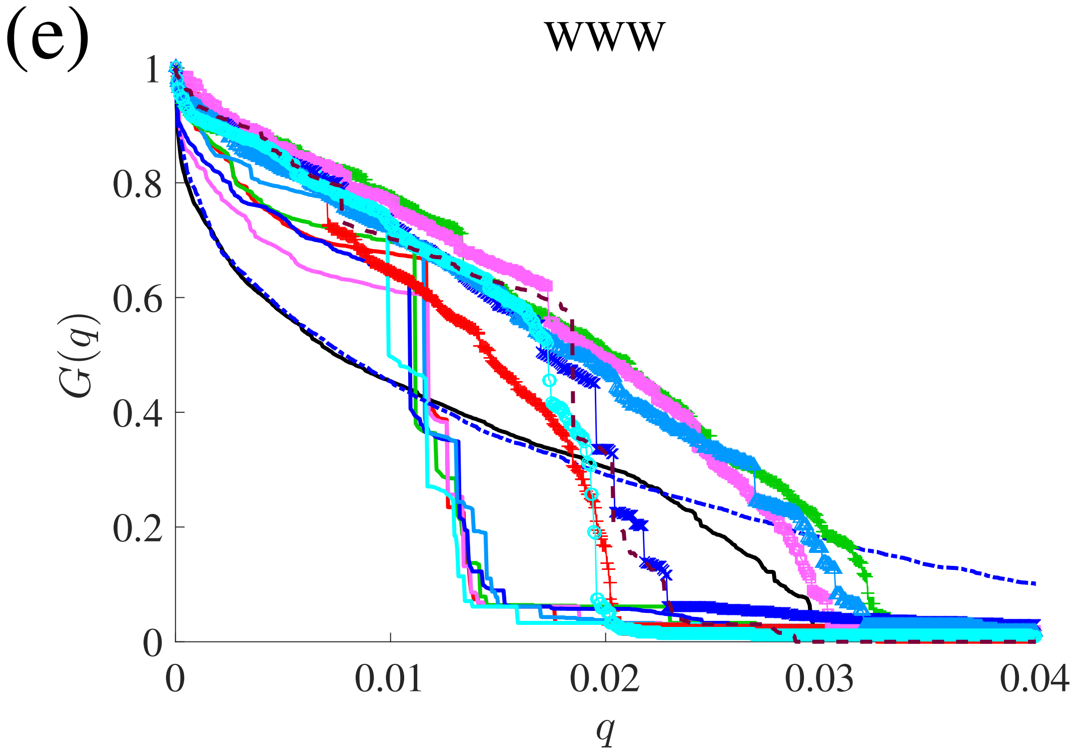
(e)
WWW
1
0.8
0.6
0.4
0.2
0
$G(q)$
0
0.01
0.02
0.03
0.04
q

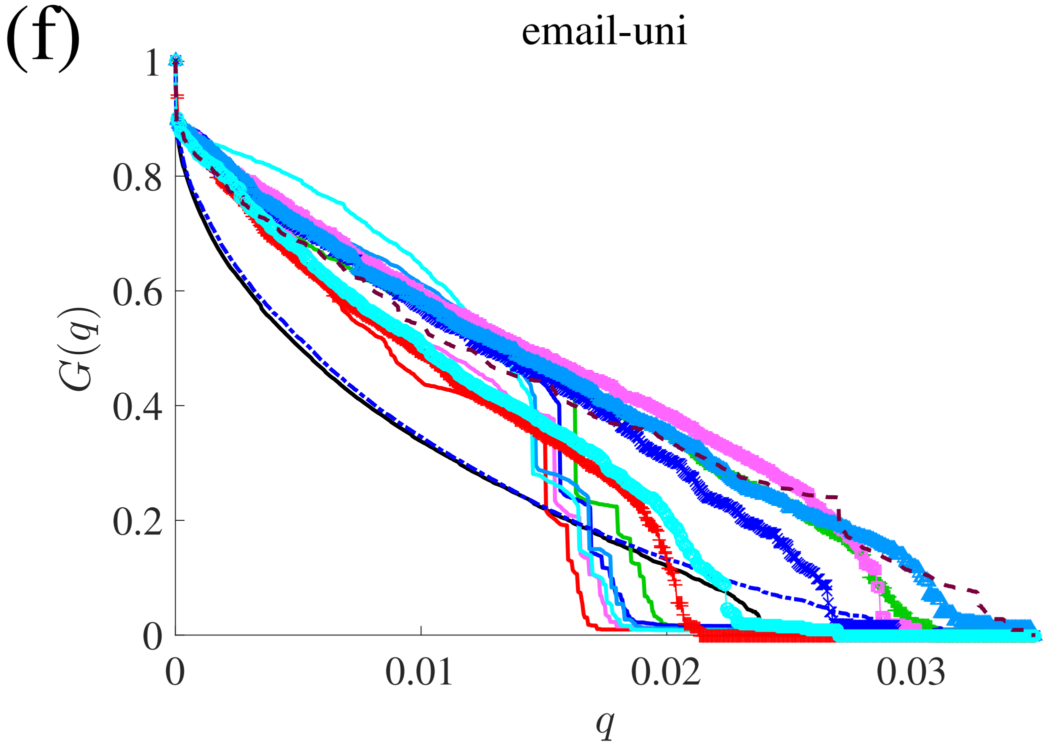
(f)
email-uni
1
0.8
0.6
0.4
0.2
0
$G(q)$
0
0.01
0.02
0.03
q

(g)

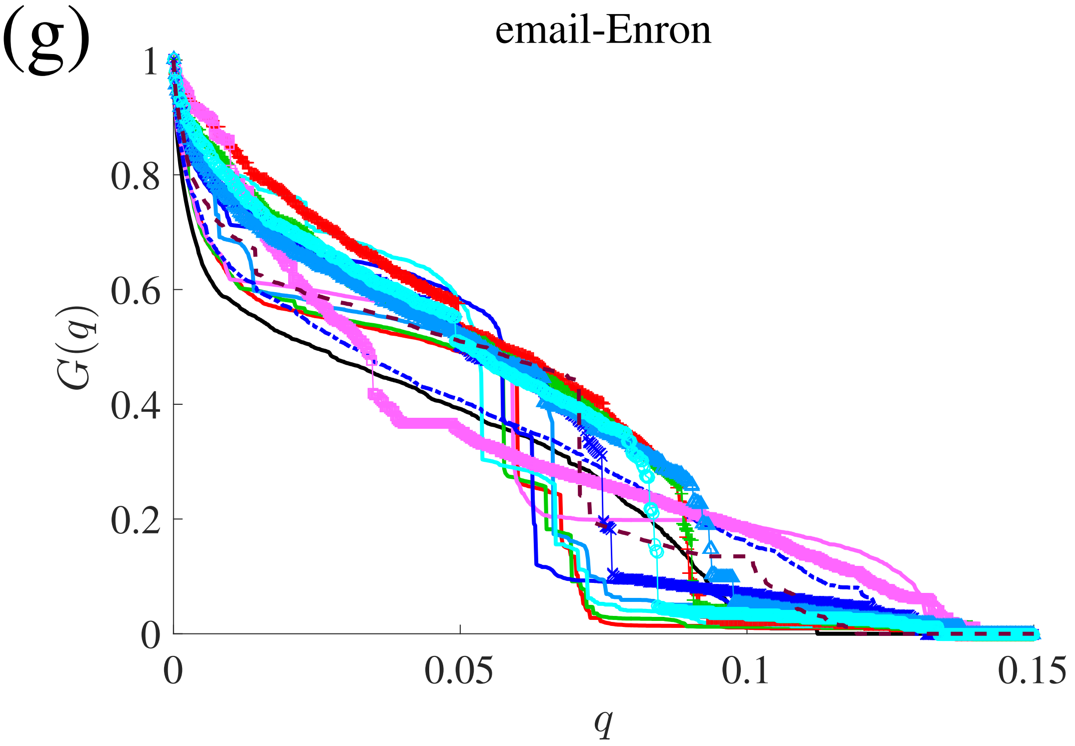
email-Enron
1
0.8
0.6
0.4
0.2
0
G(q)
0
0.05
0.1
0.15
q

(h)

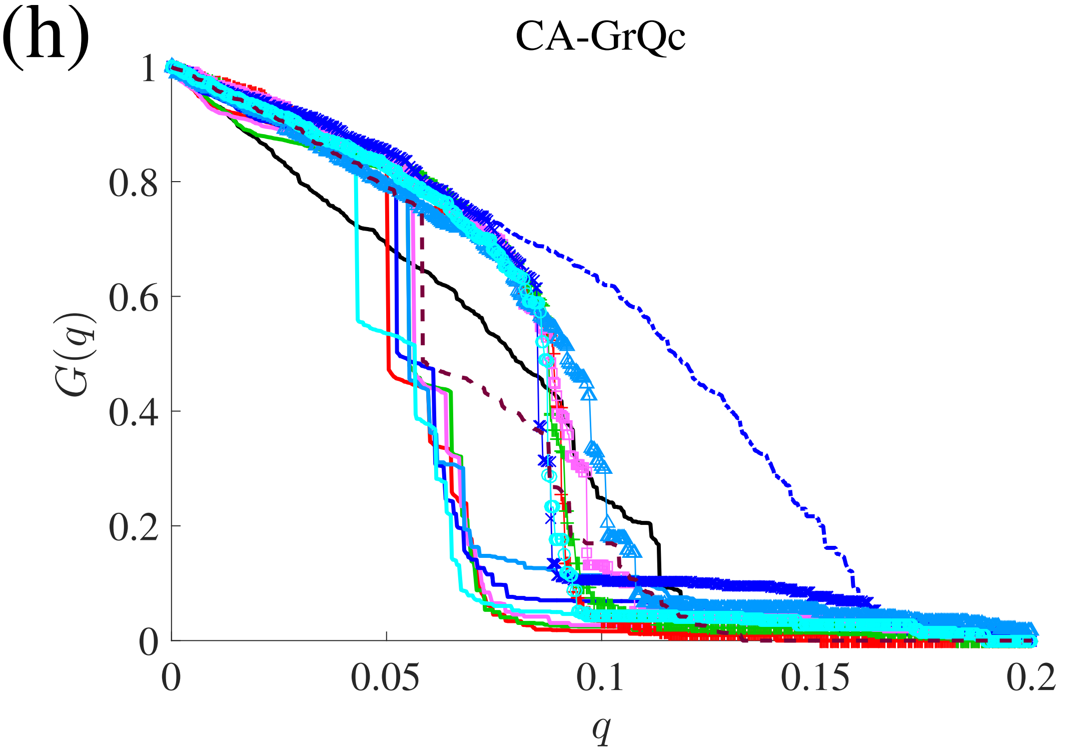
CA-GrQc
1
0.8
0.6
0.4
0.2
0
G(q)
0
0.05
0.1
0.15
0.2
q

(i)

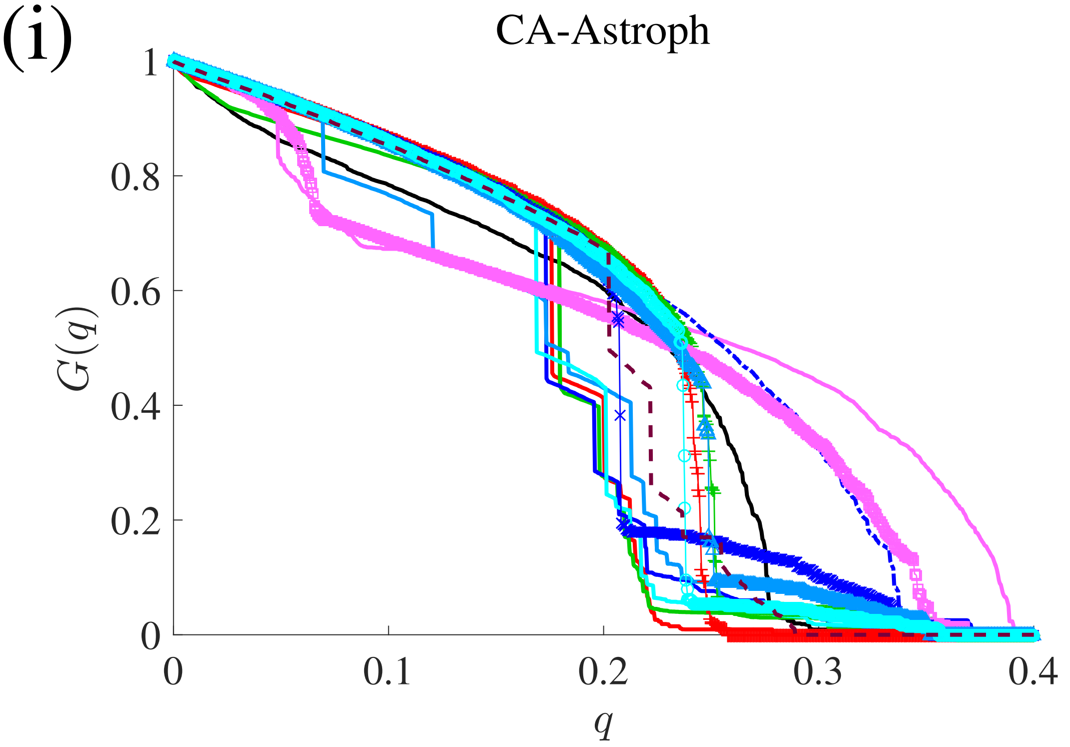
CA-Astroph
1
0.8
0.6
0.4
0.2
0
G(q)
0
0.1
0.2
0.3
0.4
q

(j)

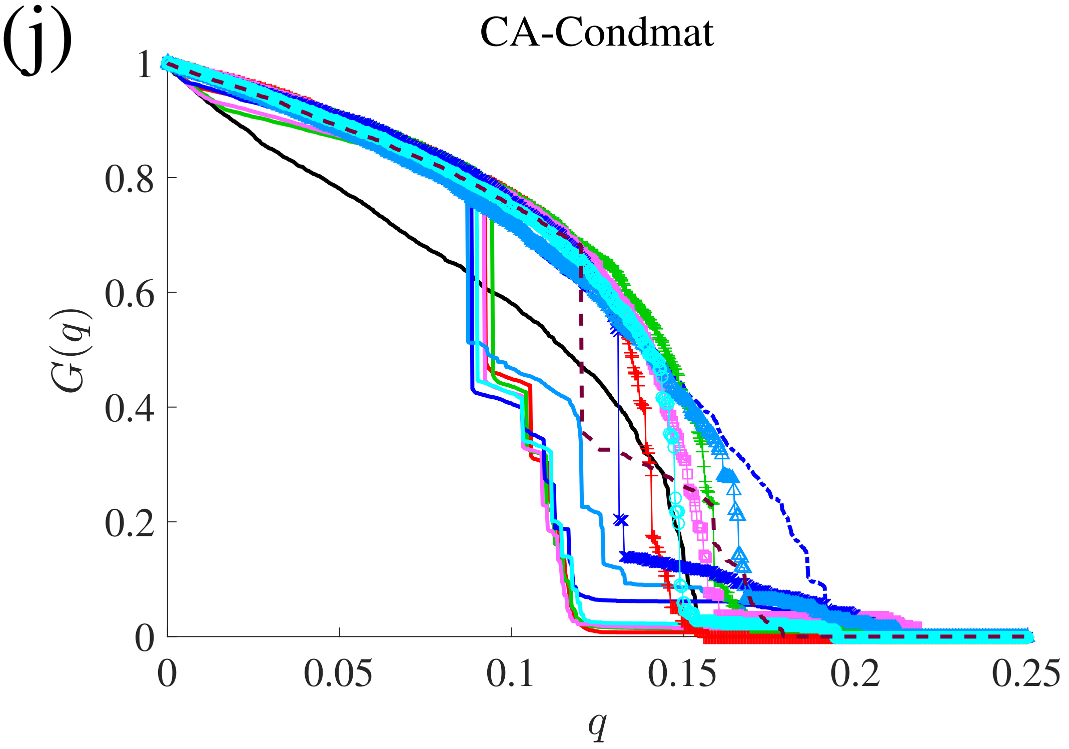
CA-Condmat
1
0.8
0.6
0.4
0.2
0
G(q)
0
0.05
0.1
0.15
0.2
0.25
q

(k)

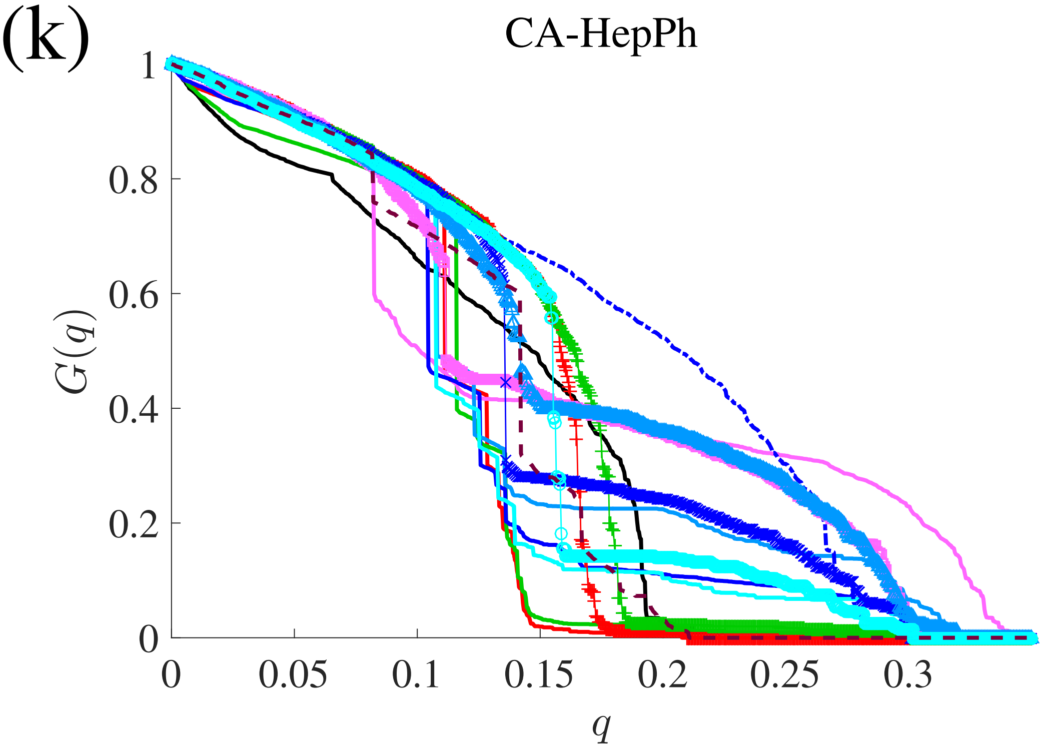
CA-HepPh
1
0.8
0.6
0.4
0.2
0
G(q)
0
0.05
0.1
0.15
0.2
0.25
0.3
q

(l)

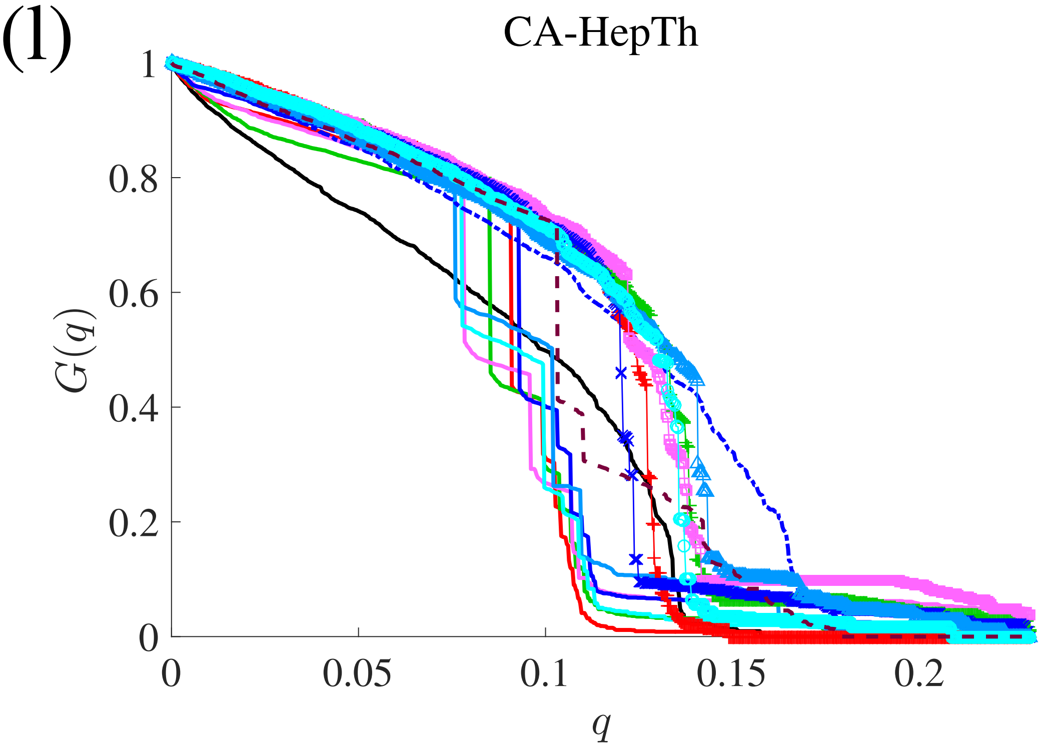
CA-HepTh
1
0.8
0.6
0.4
0.2
0
G(q)
0
0.05
0.1
0.15
0.2
q

(m)

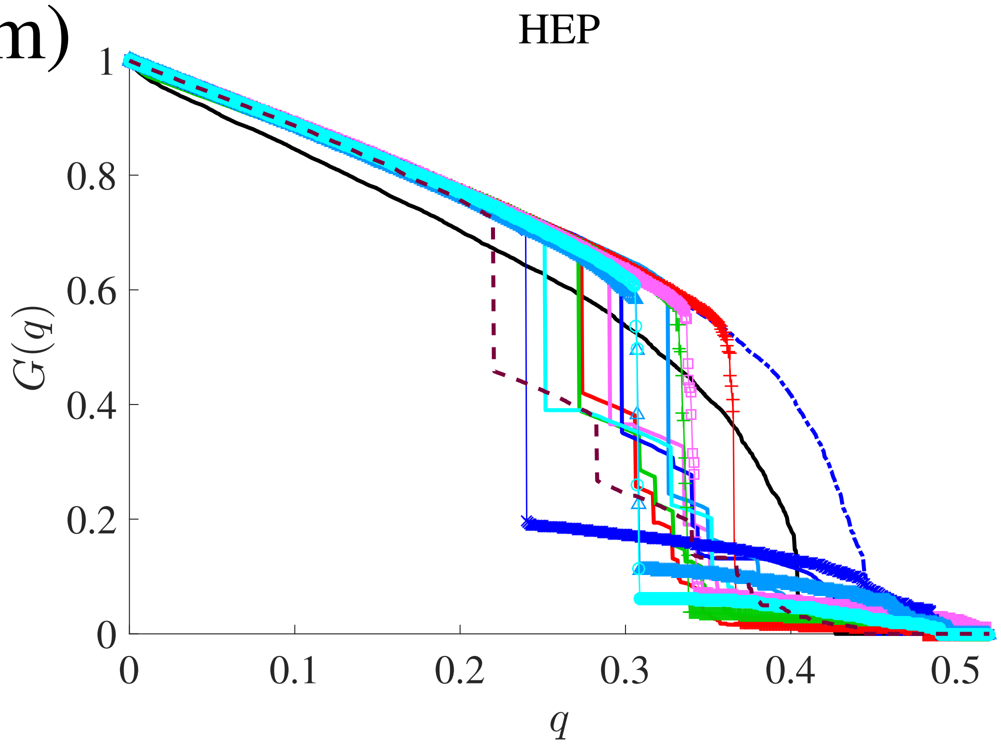

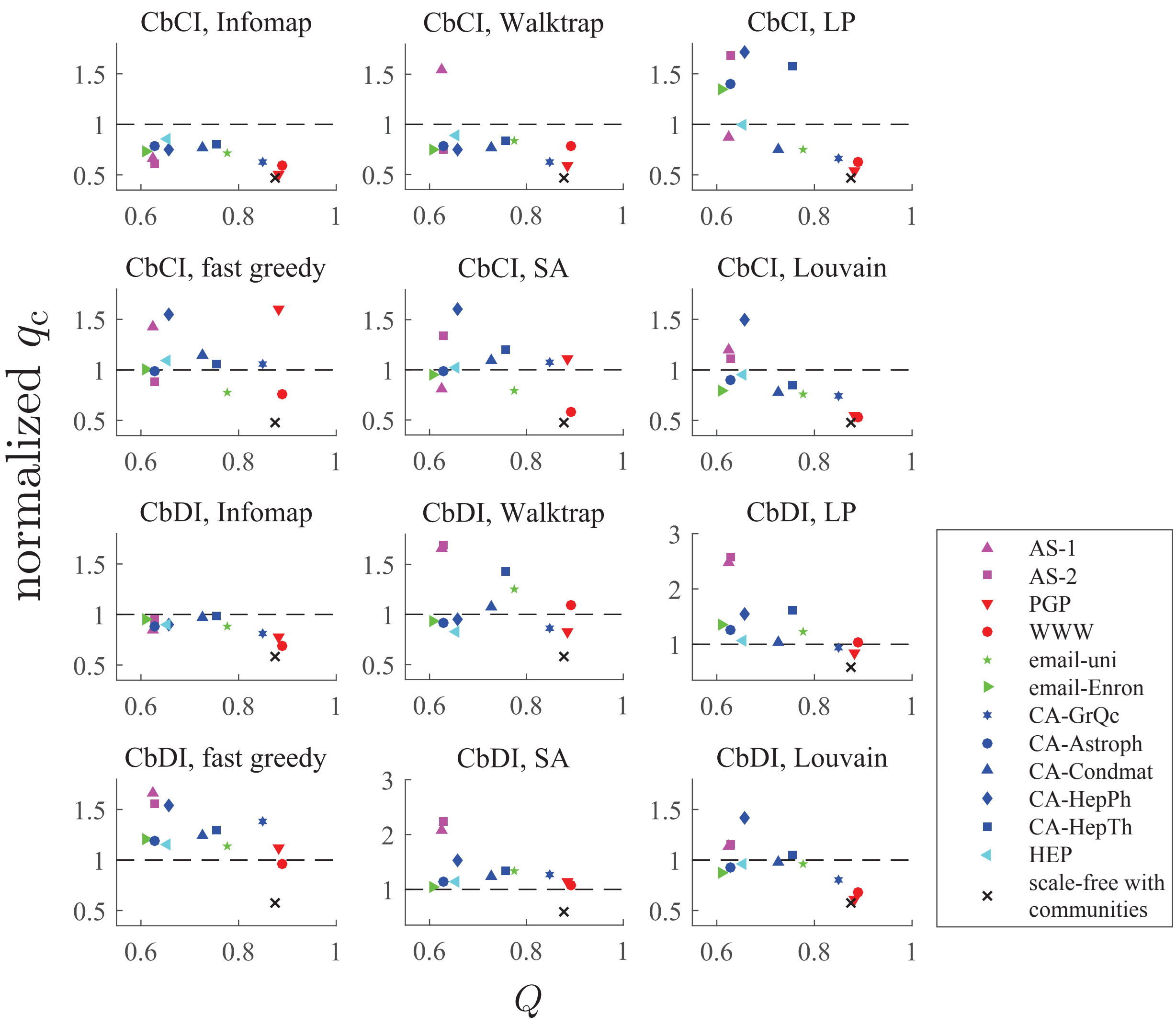

FIG. S3. Threshold fraction of removed nodes to fragment the network, q_c, for the CbCI and CbDI algorithms combined with different community detection algorithms. The shown q_c values are normalized by those for the CI algorithm. Each panel represents an immunization algorithm. A symbol represents a network. We calculated the modularity (i.e., Q) using the Louvain algorithm. The results for Infomap and Walktrap are identical to those shown in Figs. 4(a), (b), (d), and (e).

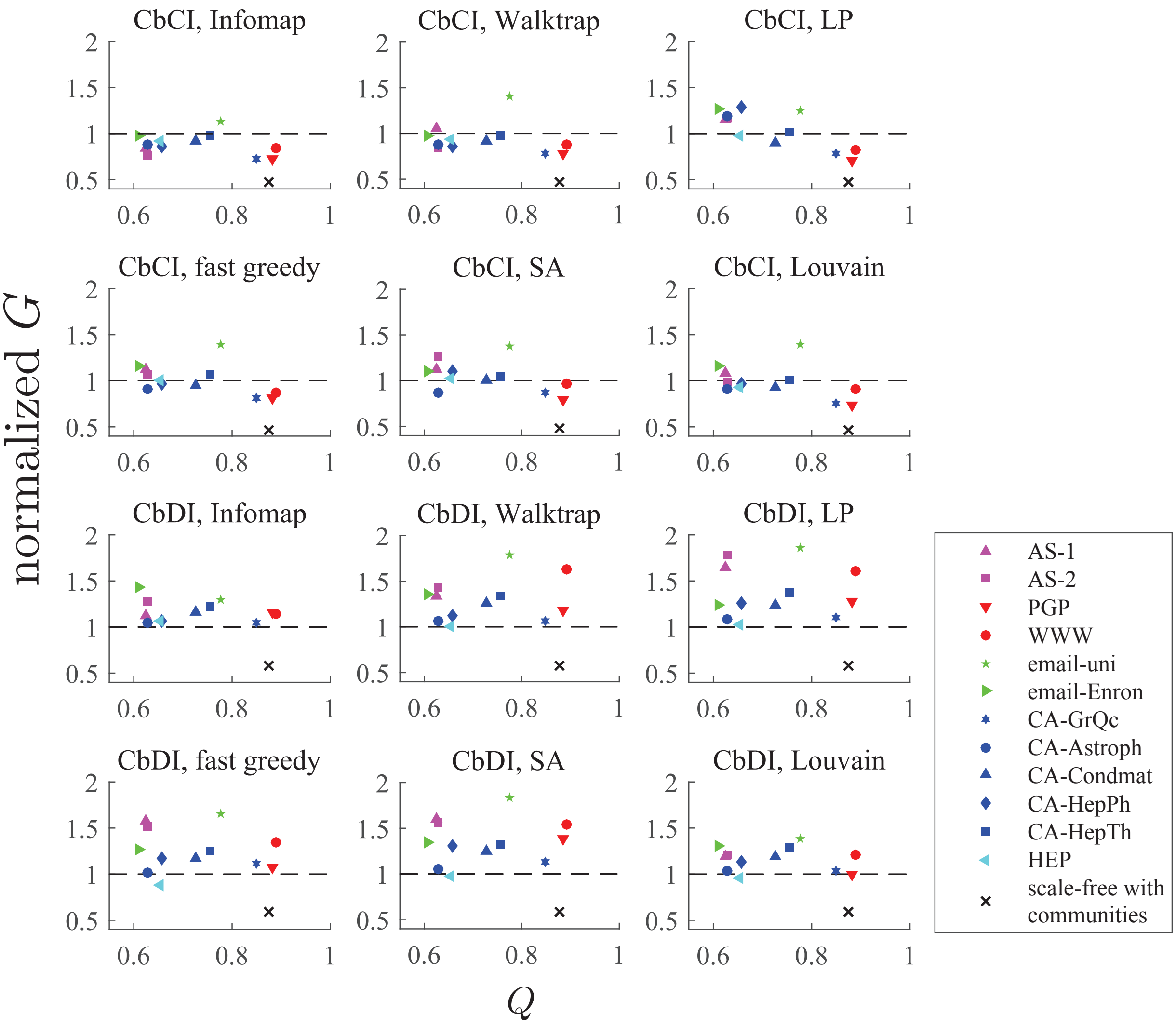

FIG. S4. The normalized size of the LCC averaged over q, i.e., $\overline{G}$, for the CbCI and CbDI algorithms. The shown values are normalized by those for the CI algorithm. See the caption of Fig. S3 for the legends. The results for Infomap and Walktrap are identical to those shown in Figs. 5(a), (b), (d), and (e).

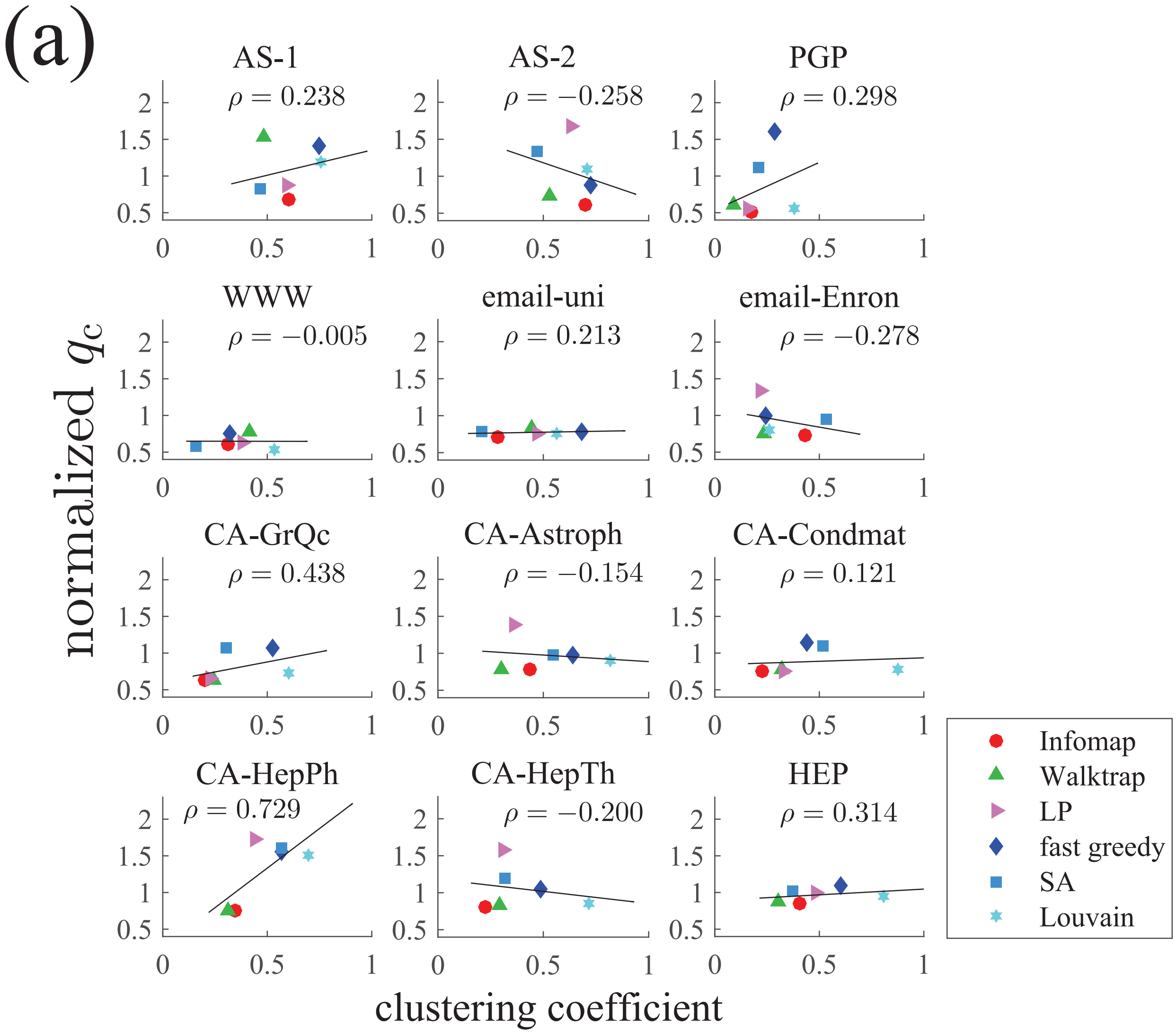

FIG. S5. Comparison of the community detection algorithms on the basis of the relationships between structural measures of the coarse-grained network and performance measures. The structural measure is calculated for each partitioning of the network. Plotted are the relationships between (a) the unweighted clustering coefficient and q_c, (b) the unweighted clustering coefficient and $\overline{G}$, (c) the weighted clustering coefficient and q_c, (d) the weighted clustering coefficient and $\overline{G}$, (e) N_c and q_c, (f) N_c and $\overline{G}$, (g) the mean path length and q_c, (h) the mean path length and $\overline{G}$, (i) the unnormalized entropy and q_c, (j) the unnormalized entropy and $\overline{G}$, (k) the normalized entropy and q_c, and (l) the normalized entropy and $\overline{G}$. The q_c and $\overline{G}$ values are those for the CbCI algorithm normalized by the values for the CI algorithm.

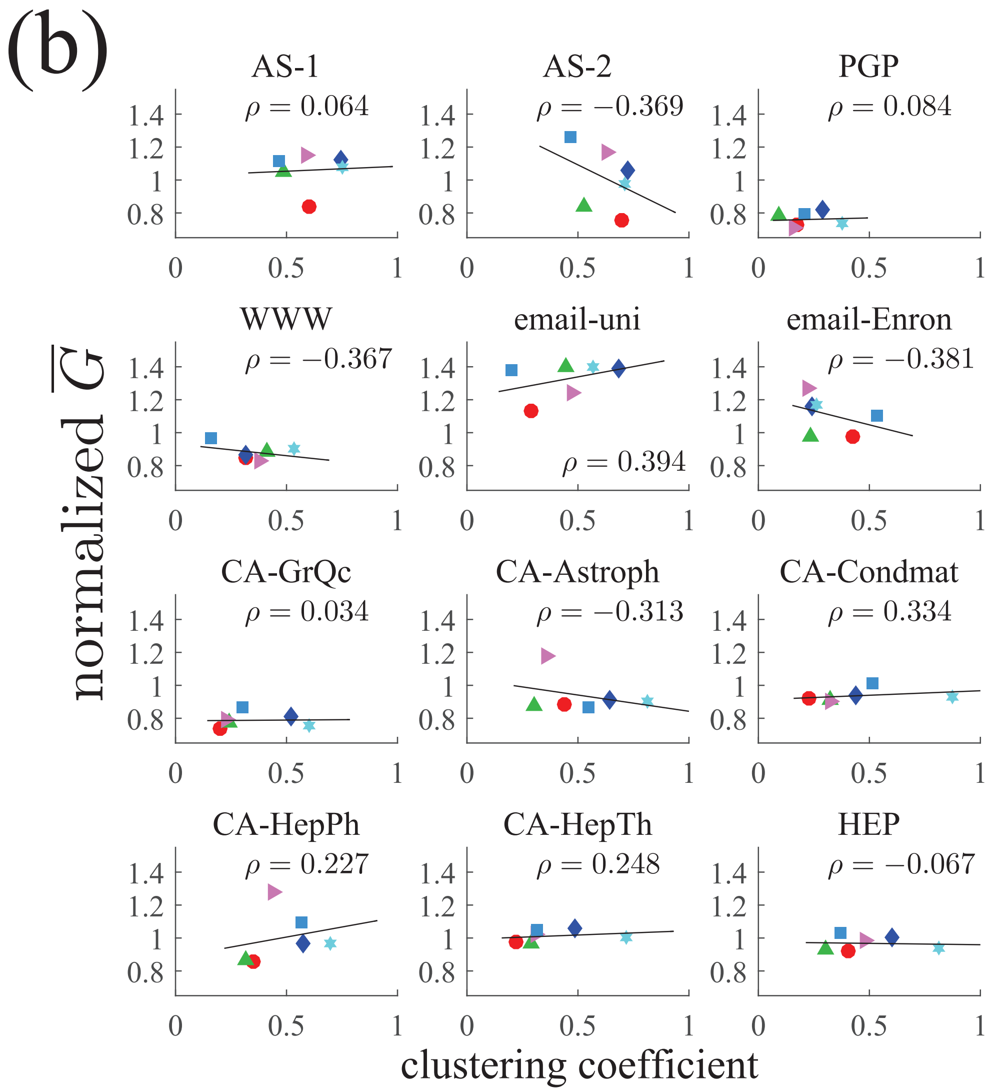
(b)
AS-1
ρ = 0.064
AS-2
ρ = −0.369
PGP
ρ = 0.084
WWW
ρ = −0.367
email-uni
ρ = 0.394
email-Enron
ρ = −0.381
CA-GrQc
ρ = 0.034
CA-Astroph
ρ = −0.313
CA-Condmat
ρ = 0.334
CA-HepPh
ρ = 0.227
CA-HepTh
ρ = 0.248
HEP
ρ = −0.067
normalized $\overline{G}$
clustering coefficient

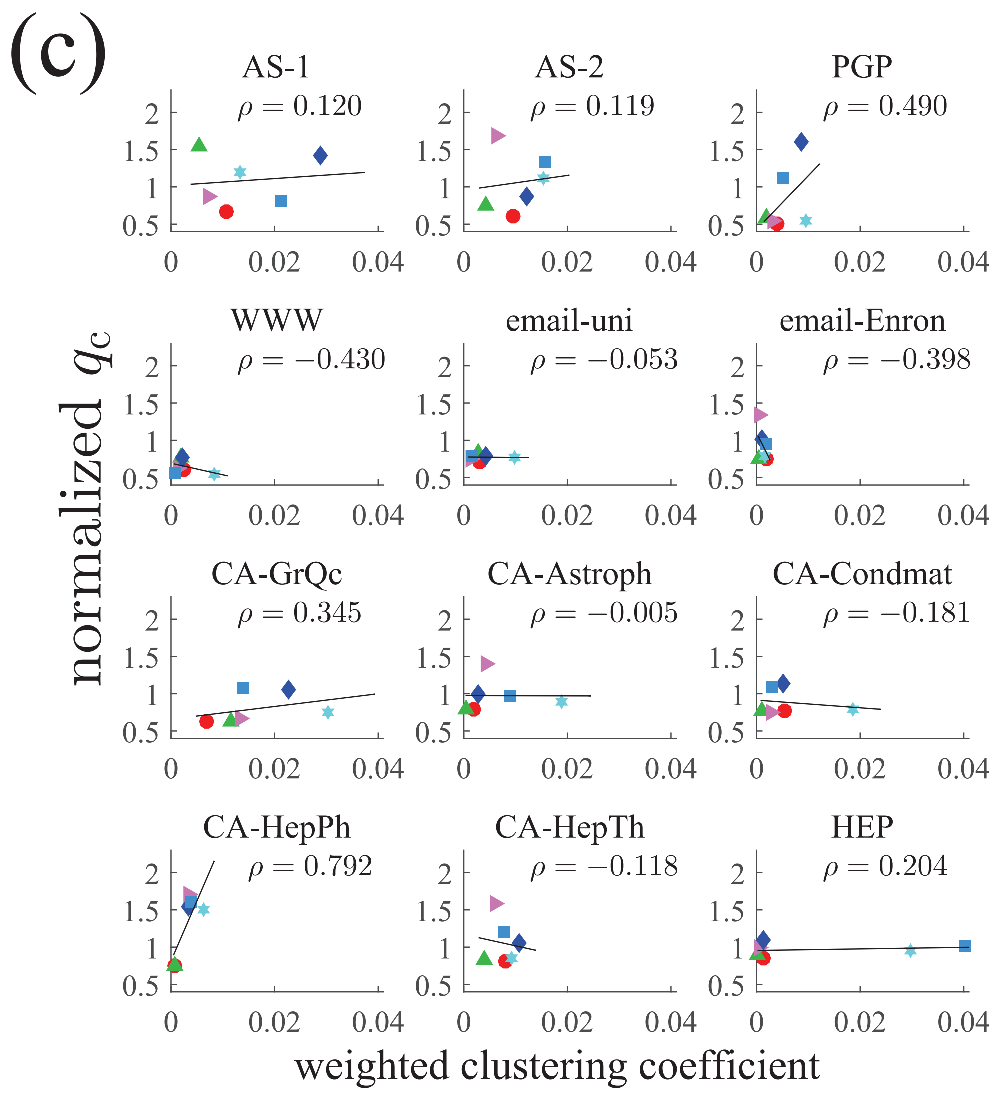
(c)
AS-1
$\rho = 0.120$
AS-2
$\rho = 0.119$
PGP
$\rho = 0.490$
WWW
$\rho = -0.430$
email-uni
$\rho = -0.053$
email-Enron
$\rho = -0.398$
CA-GrQc
$\rho = 0.345$
CA-Astroph
$\rho = -0.005$
CA-Condmat
$\rho = -0.181$
CA-HepPh
$\rho = 0.792$
CA-HepTh
$\rho = -0.118$
HEP
$\rho = 0.204$
normalized q_c
weighted clustering coefficient
0.5
1
1.5
2
0
0.02
0.04

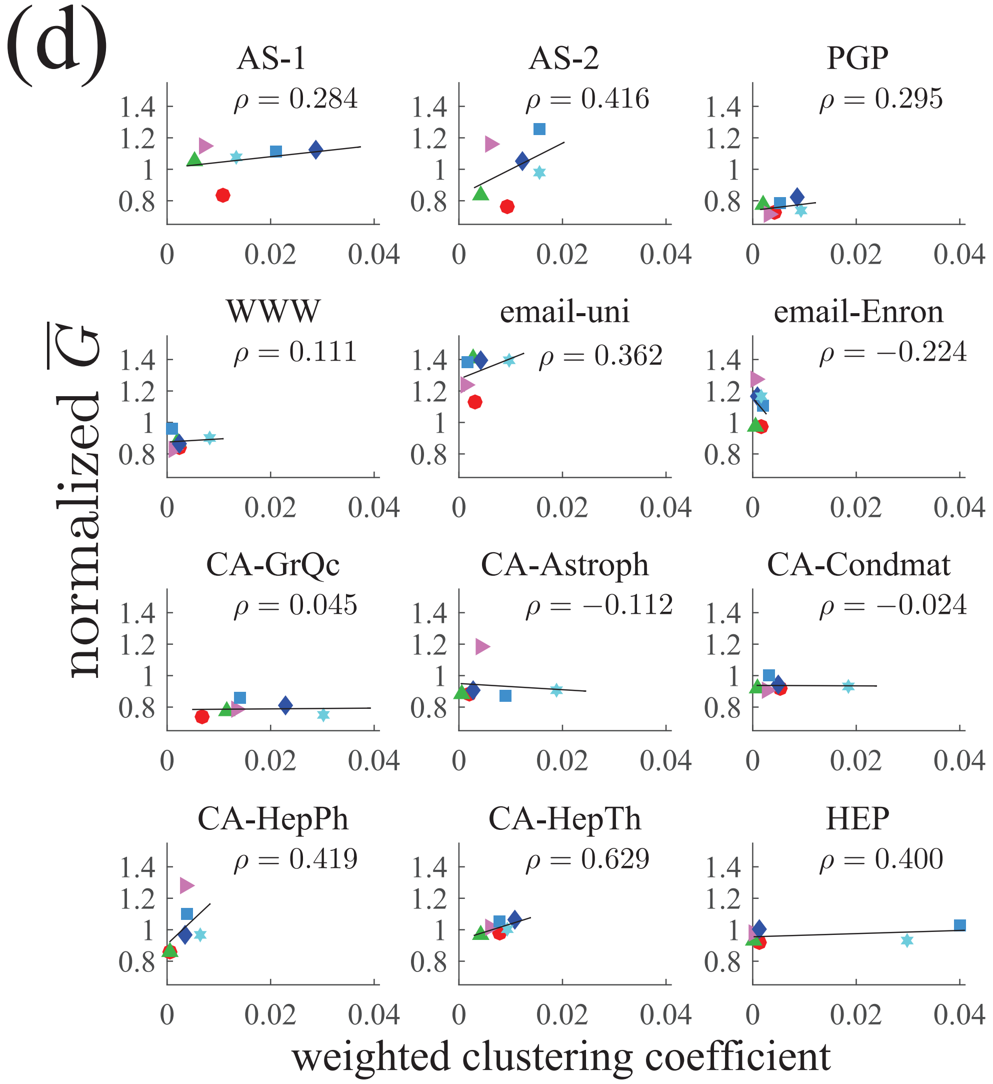
(d)
AS-1
ρ = 0.284
AS-2
ρ = 0.416
PGP
ρ = 0.295
WWW
ρ = 0.111
email-uni
ρ = 0.362
email-Enron
ρ = −0.224
CA-GrQc
ρ = 0.045
CA-Astroph
ρ = −0.112
CA-Condmat
ρ = −0.024
CA-HepPh
ρ = 0.419
CA-HepTh
ρ = 0.629
HEP
ρ = 0.400
normalized $\overline{G}$
weighted clustering coefficient
1.4
1.2
1
0.8
0
0.02
0.04

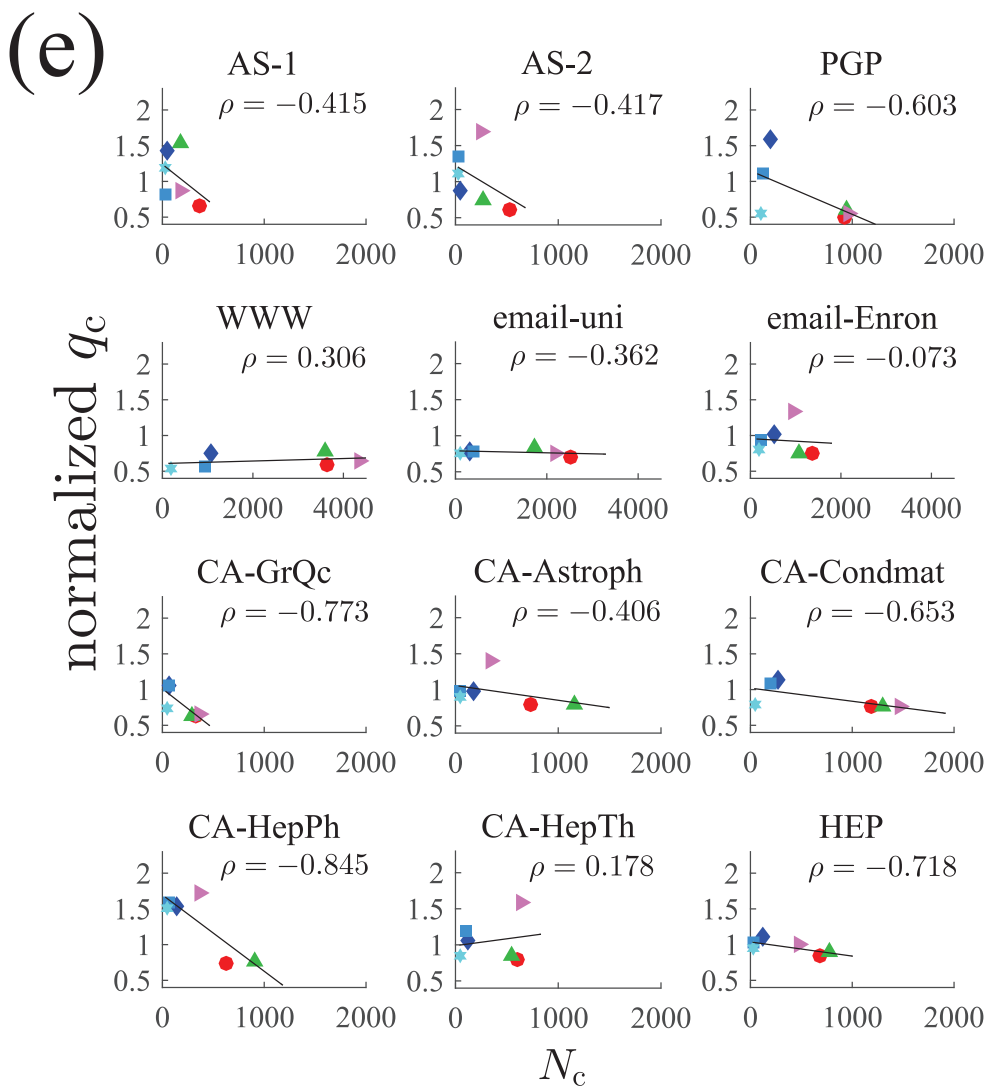
(e)
AS-1
ρ = −0.415
AS-2
ρ = −0.417
PGP
ρ = −0.603
WWW
ρ = 0.306
email-uni
ρ = −0.362
email-Enron
ρ = −0.073
CA-GrQc
ρ = −0.773
CA-Astroph
ρ = −0.406
CA-Condmat
ρ = −0.653
CA-HepPh
ρ = −0.845
CA-HepTh
ρ = 0.178
HEP
ρ = −0.718
normalized q_c
N_c

(f)

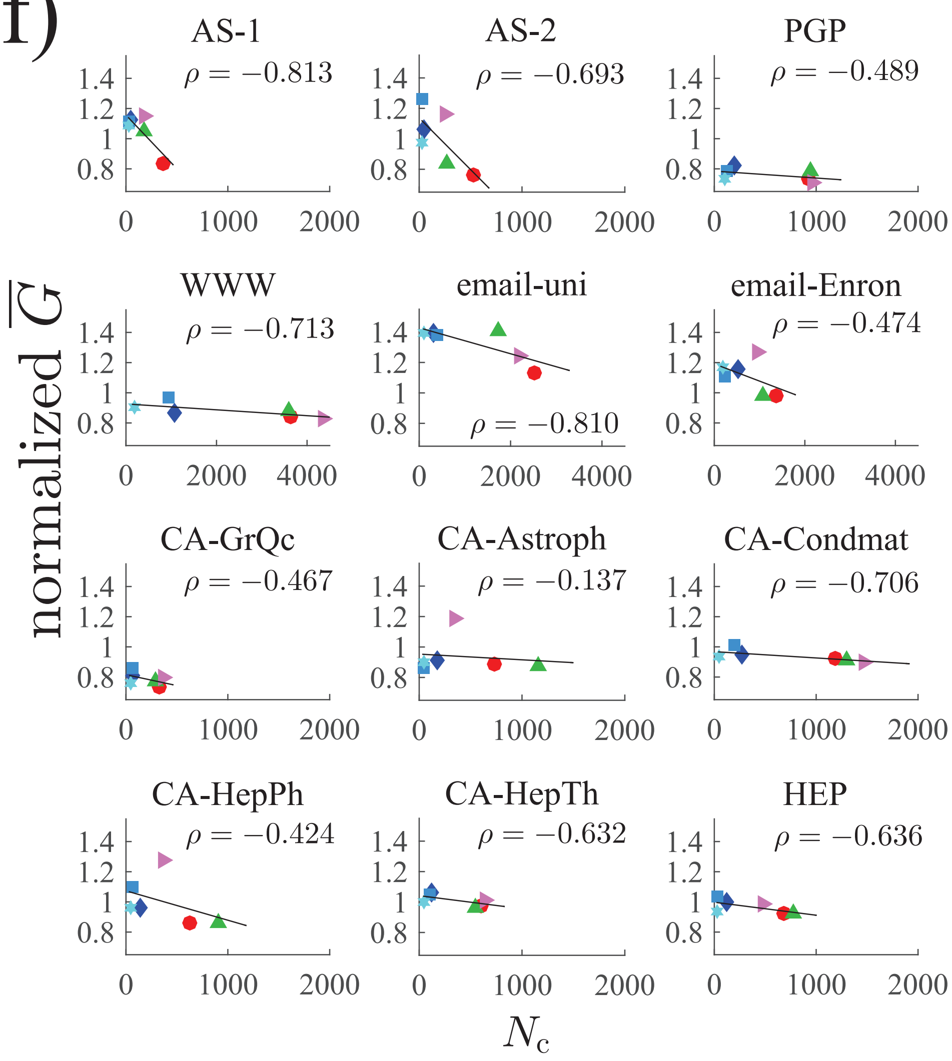
AS-1
ρ = −0.813
AS-2
ρ = −0.693
PGP
ρ = −0.489
WWW
ρ = −0.713
email-uni
ρ = −0.810
email-Enron
ρ = −0.474
CA-GrQc
ρ = −0.467
CA-Astroph
ρ = −0.137
CA-Condmat
ρ = −0.706
CA-HepPh
ρ = −0.424
CA-HepTh
ρ = −0.632
HEP
ρ = −0.636
normalized $\overline{G}$
N_c

(g)

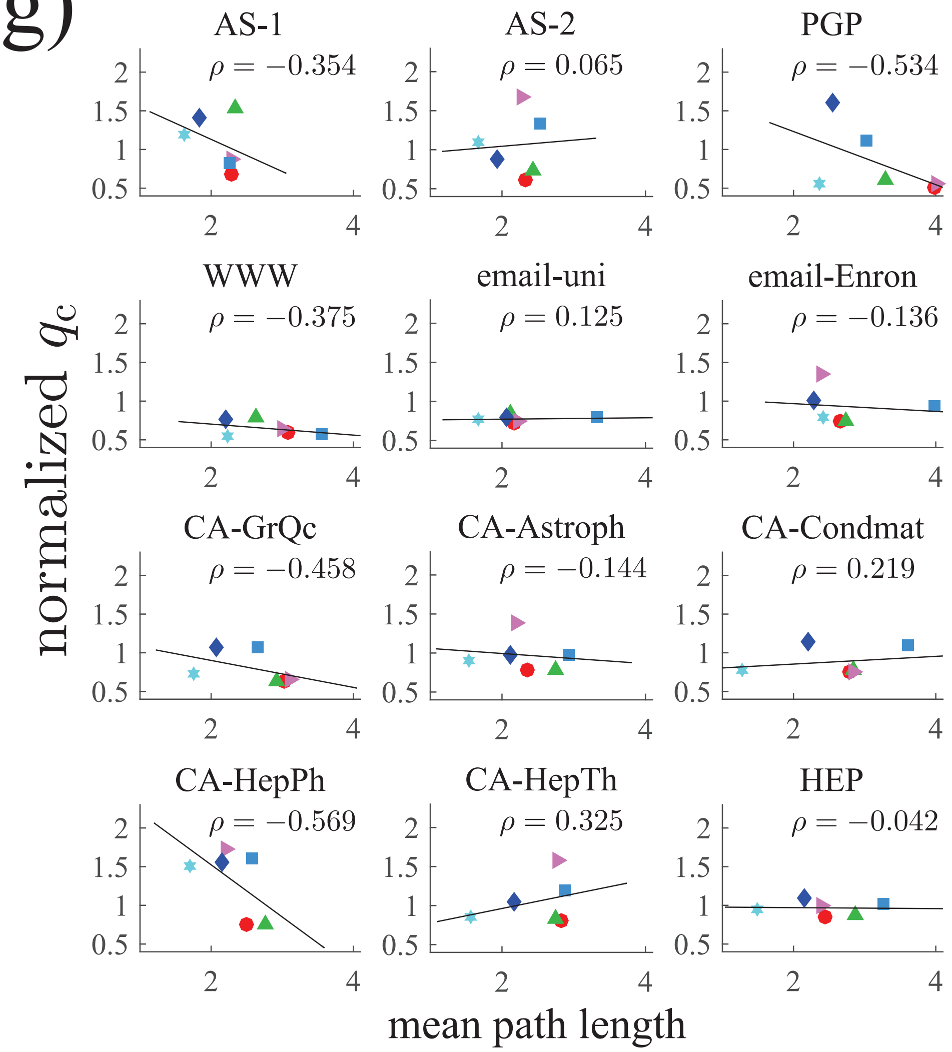
AS-1
$\rho = -0.354$
AS-2
$\rho = 0.065$
PGP
$\rho = -0.534$
WWW
$\rho = -0.375$
email-uni
$\rho = 0.125$
email-Enron
$\rho = -0.136$
CA-GrQc
$\rho = -0.458$
CA-Astroph
$\rho = -0.144$
CA-Condmat
$\rho = 0.219$
CA-HepPh
$\rho = -0.569$
CA-HepTh
$\rho = 0.325$
HEP
$\rho = -0.042$
normalized q_c
mean path length

(h)

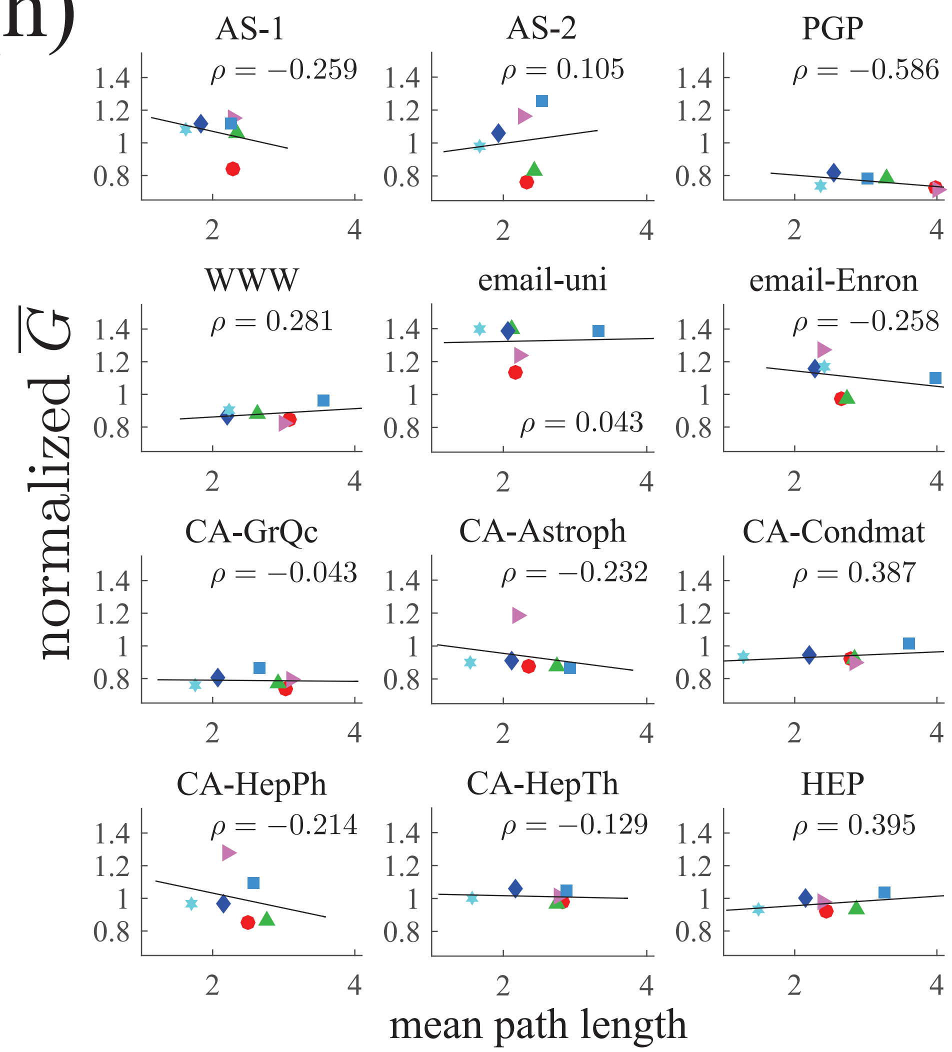
AS-1
$\rho = -0.259$
AS-2
$\rho = 0.105$
PGP
$\rho = -0.586$
WWW
$\rho = 0.281$
email-uni
$\rho = 0.043$
email-Enron
$\rho = -0.258$
CA-GrQc
$\rho = -0.043$
CA-Astroph
$\rho = -0.232$
CA-Condmat
$\rho = 0.387$
CA-HepPh
$\rho = -0.214$
CA-HepTh
$\rho = -0.129$
HEP
$\rho = 0.395$
normalized $\overline{G}$
mean path length

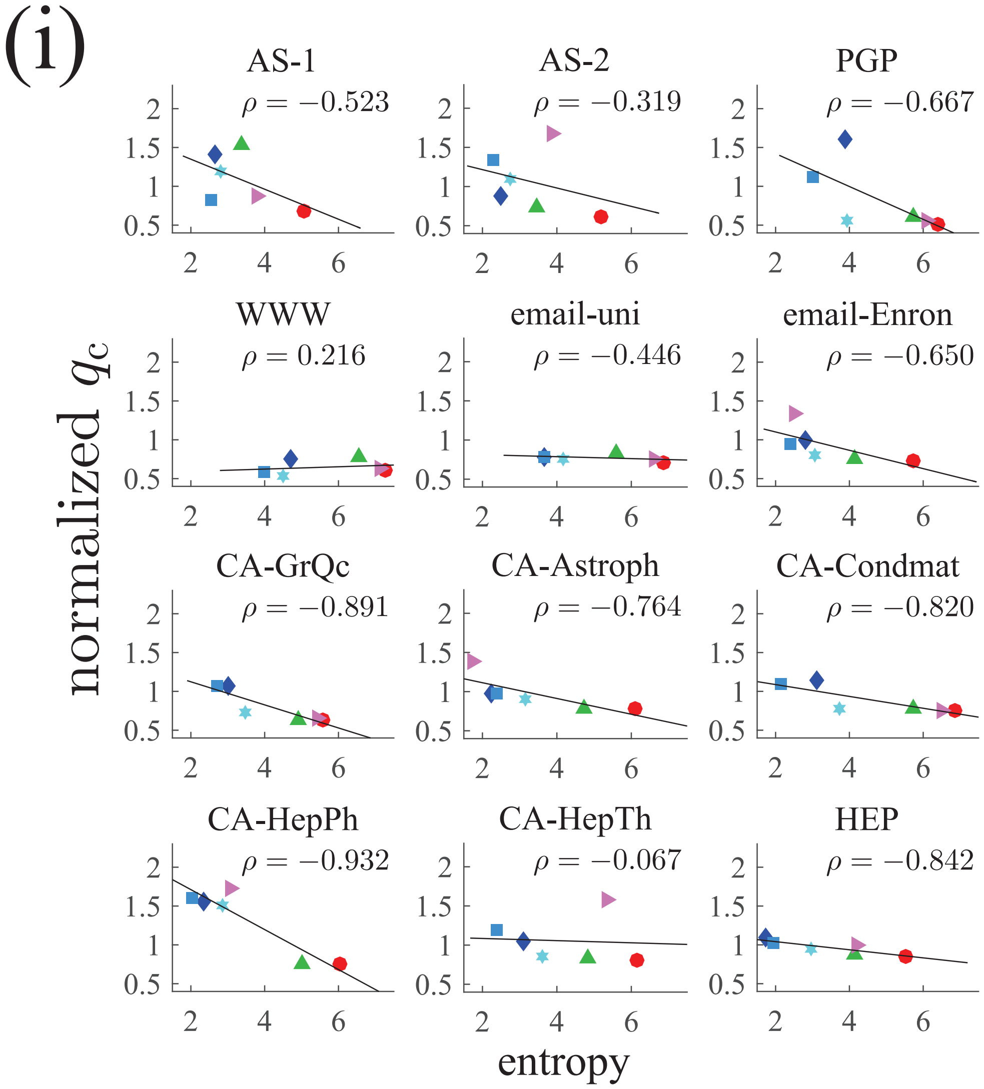
(i)
AS-1
ρ = −0.523
AS-2
ρ = −0.319
PGP
ρ = −0.667
WWW
ρ = 0.216
email-uni
ρ = −0.446
email-Enron
ρ = −0.650
CA-GrQc
ρ = −0.891
CA-Astroph
ρ = −0.764
CA-Condmat
ρ = −0.820
CA-HepPh
ρ = −0.932
CA-HepTh
ρ = −0.067
HEP
ρ = −0.842
normalized qc
entropy

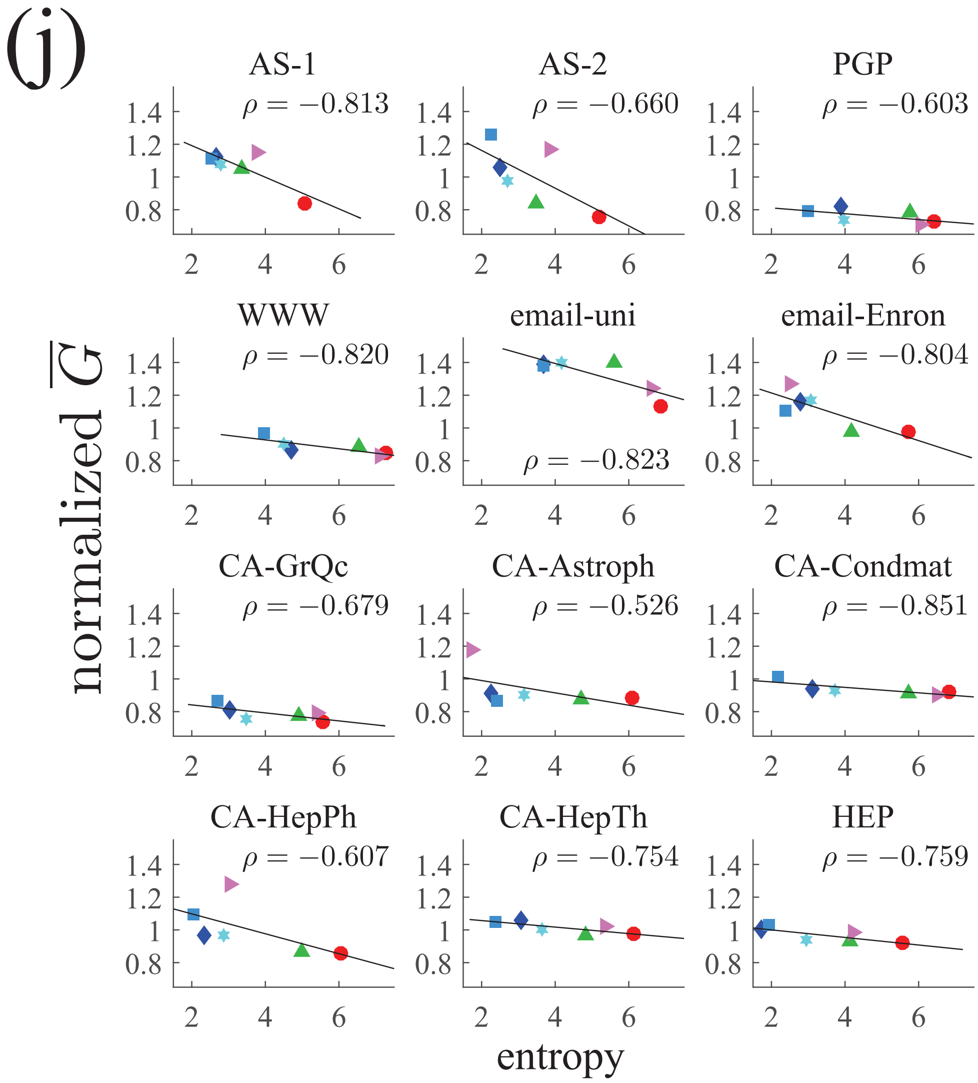
(j)
AS-1
ρ = −0.813
AS-2
ρ = −0.660
PGP
ρ = −0.603
WWW
ρ = −0.820
email-uni
ρ = −0.823
email-Enron
ρ = −0.804
CA-GrQc
ρ = −0.679
CA-Astroph
ρ = −0.526
CA-Condmat
ρ = −0.851
CA-HepPh
ρ = −0.607
CA-HepTh
ρ = −0.754
HEP
ρ = −0.759
normalized $\overline{G}$
entropy

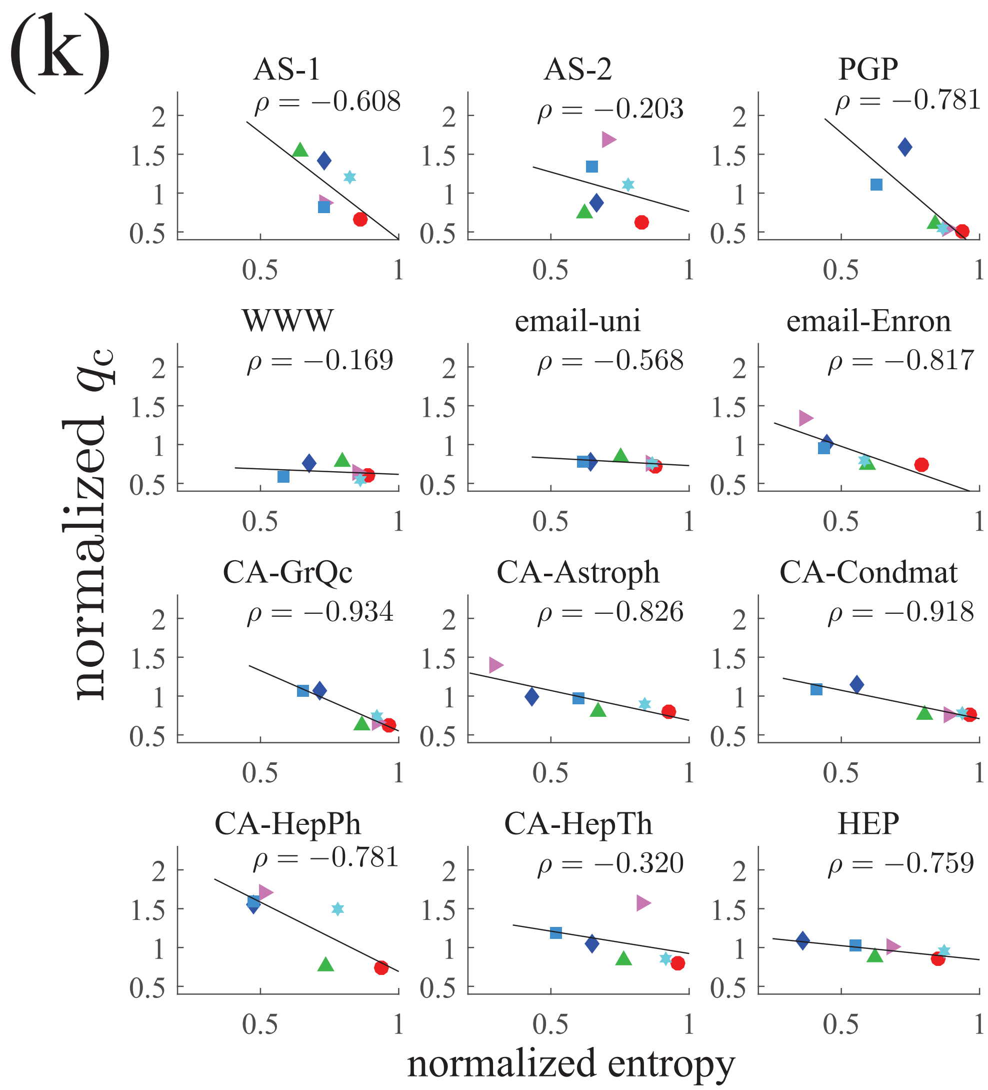
(k)
AS-1
ρ = −0.608
AS-2
ρ = −0.203
PGP
ρ = −0.781
WWW
ρ = −0.169
email-uni
ρ = −0.568
email-Enron
ρ = −0.817
CA-GrQc
ρ = −0.934
CA-Astroph
ρ = −0.826
CA-Condmat
ρ = −0.918
CA-HepPh
ρ = −0.781
CA-HepTh
ρ = −0.320
HEP
ρ = −0.759
normalized q_c
normalized entropy

(l)

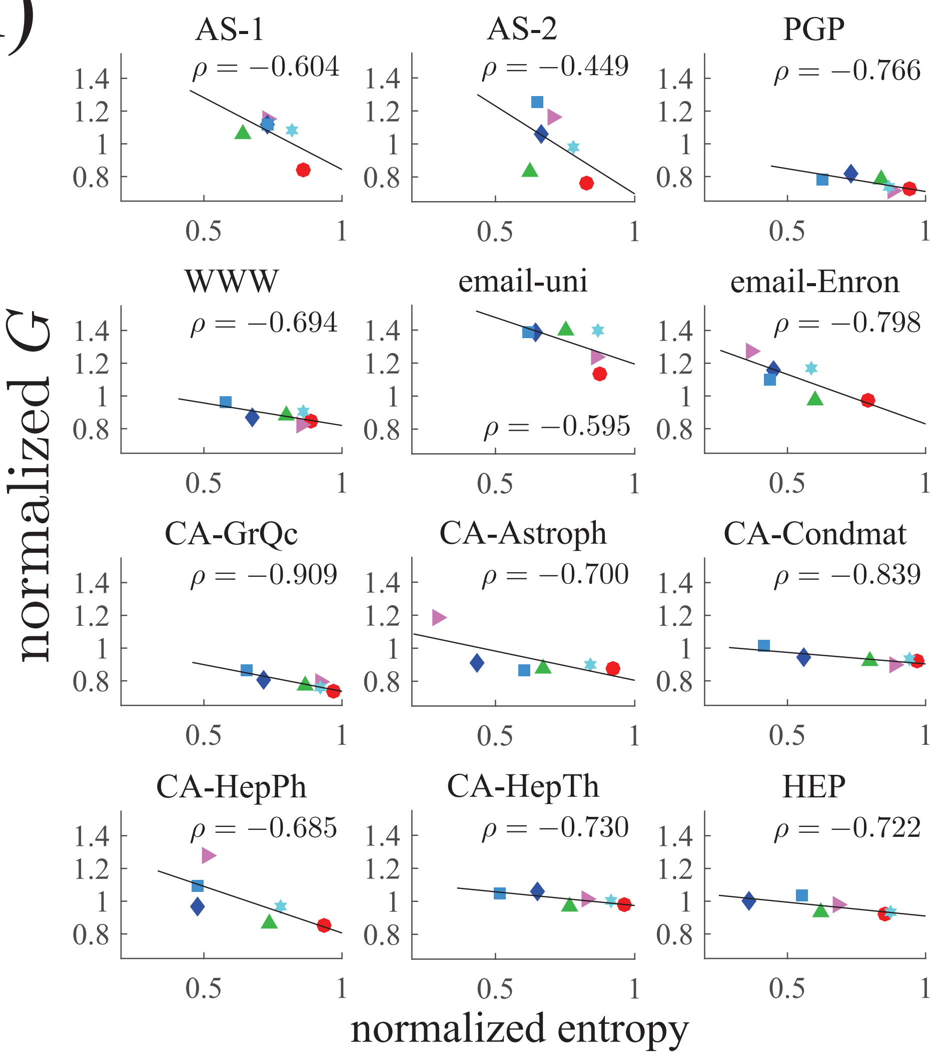

AS-1
ρ = −0.604
AS-2
ρ = −0.449
PGP
ρ = −0.766
WWW
ρ = −0.694
email-uni
ρ = −0.595
email-Enron
ρ = −0.798
CA-GrQc
ρ = −0.909
CA-Astroph
ρ = −0.700
CA-Condmat
ρ = −0.839
CA-HepPh
ρ = −0.685
CA-HepTh
ρ = −0.730
HEP
ρ = −0.722
normalized G
normalized entropy

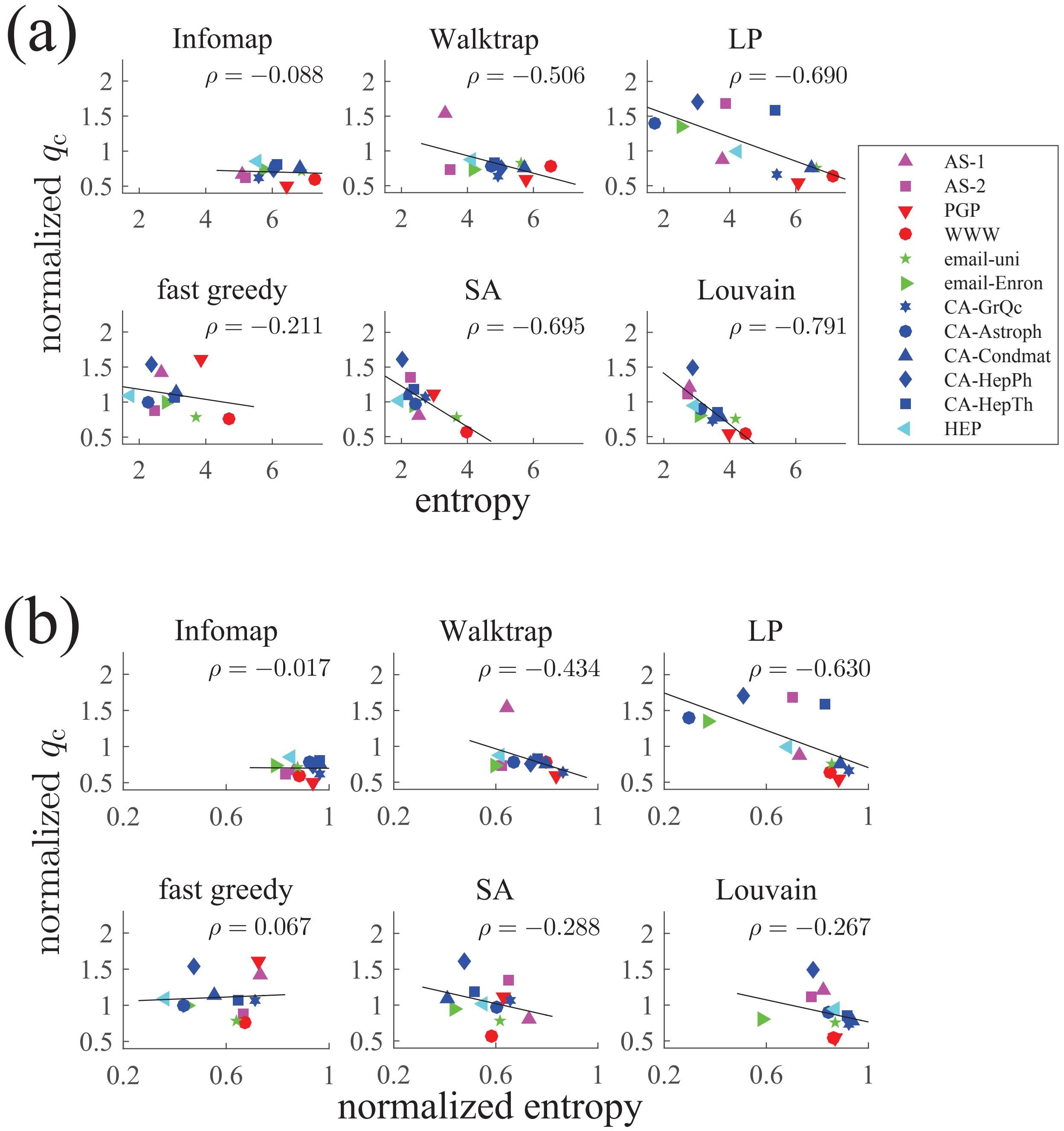

FIG. S6. Comparison of the different networks using the relationship between the entropy and performance measures. (a) Relationship between the unnormalized entropy and the normalized q_c. (b) Relationship between the normalized entropy and the normalized q_c. (c) Relationship between the unnormalized entropy and the normalized $\overline{G}$. (d) Relationship between the normalized entropy and the normalized $\overline{G}$.

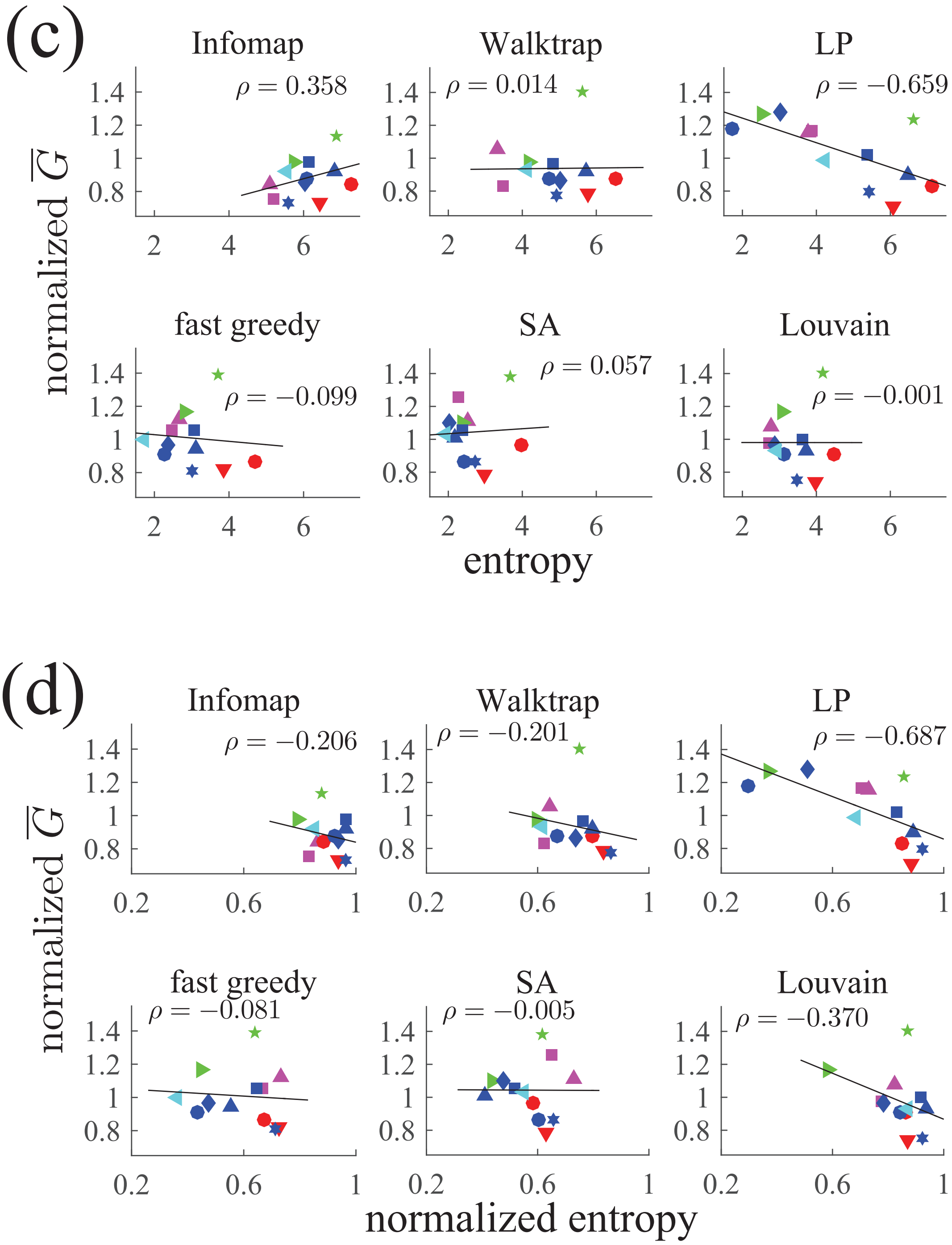
(c)
Infomap
Walktrap
LP
fast greedy
SA
Louvain
ρ = 0.358
ρ = 0.014
ρ = −0.659
ρ = −0.099
ρ = 0.057
ρ = −0.001
normalized $\overline{G}$
entropy
(d)
Infomap
Walktrap
LP
fast greedy
SA
Louvain
ρ = −0.206
ρ = −0.201
ρ = −0.687
ρ = −0.081
ρ = −0.005
ρ = −0.370
normalized $\overline{G}$
normalized entropy

TABLE S1. Summary statistics of the model and empirical networks. N, M, and $\langle k \rangle$ represent the number of nodes, that of links, and the average degree, respectively.

Network	N	M	$\langle k \rangle$	References
scale-free with communities	5,000	21,440	8.58	[1]
BA	5,000	29,979	11.99	[2]
AS-1	6,474	12,572	3.88	[3, 4]
AS-2	10,670	22,002	4.12	[3, 4]
PGP	10,680	24,316	4.55	[5]
WWW	99,193	178,840	3.61	[6]
email-uni	63,495	96,777	3.05	[7]
email-Enron	33,696	180,811	10.73	[4, 8–10]
CA-GrQc	4,158	13,422	6.46	[4, 11]
CA-Astroph	17,903	196,972	22.00	[4, 11]
CA-Condmat	21,363	91,286	8.55	[4, 11]
CA-HepPh	11,204	117,619	21.00	[3, 4, 12]
CA-HepTh	8,638	24,806	5.74	[3, 4, 12]
HEP	27,400	352,021	25.69	[13]

TABLE S2. Community structure of the model and empirical networks detected by different algorithms. The number of communities, N_{C}, and the modularity, Q, are shown for each community detection algorithm. LP: label propagation. SA: simulated annealing. We implemented the Infomap, label propagation, and simulated annealing algorithm using the codes available at [14], the Walktrap using the codes available at [15], the fast greedy algorithm using the codes available at [16], and the Louvain algorithm using the codes available at [17].

	Infomap		Walktrap		LP		fast greedy		SA		Louvain	
Network	N_{C}	Q	N_{C}	Q	N_{C}	Q	N_{C}	Q	N_{C}	Q	N_{C}	Q
BA	256	0.174	219	0.166	0	n.a.	12	0.250	19	0.250	13	0.253
AS-1	358	0.551	184	0.599	172	0.552	38	0.603	33	0.590	30	0.626
AS-2	524	0.544	262	0.599	241	0.549	42	0.614	33	0.579	33	0.629
PGP	924	0.813	946	0.830	959	0.817	205	0.853	118	0.812	96	0.883
WWW	3,643	0.823	3,607	0.805	4,334	0.815	1,067	0.850	936	0.822	186	0.890
email-uni	2,534	0.705	1,731	0.716	2,183	0.689	310	0.728	389	0.718	121	0.777
email-Enron	1,385	0.544	1,056	0.544	958	0.324	525	0.511	235	0.585	191	0.608
CA-GrQc	323	0.785	290	0.799	358	0.780	70	0.796	63	0.792	43	0.848
CA-Astroph	735	0.561	1,154	0.542	344	0.292	183	0.492	55	0.586	42	0.627
CA-Condmat	1,186	0.646	1,302	0.627	1,475	0.634	266	0.628	189	0.600	53	0.726
CA-HepPh	627	0.612	910	0.596	373	0.451	138	0.583	74	0.628	39	0.658
CA-HepTh	596	0.680	549	0.676	640	0.666	115	0.703	101	0.661	53	0.755
HEP	674	0.579	773	0.608	477	0.613	120	0.525	33	0.606	29	0.653

[1] Masuda, N. Immunization of networks with community structure. *New J. Phys.* **11**(12), 123018 (2009).

[2] Barabási, A.-L. and Albert, R. Emergence of scaling in random networks. *Science* **286**(5439), 509–512 (1999).

[3] Leskovec, J., Kleinberg, J., and Faloutsos, C. Graphs over time: densification laws, shrinking diameters and possible explanations. In *Proceedings of the Eleventh ACM SIGKDD International Conference on Knowledge Discovery in Data Mining*, 177–187. ACM, (2005).

[4] http://snap.stanford.edu/.

[5] Boguñá, M., Pastor-Satorras, R., Díaz-Guilera, A., and Arenas, A. Models of social networks based on social distance attachment. *Phys. Rev. E* **70**, 056122, Nov (2004).

[6] Albert, R., Jeong, H., and Barabási, A.-L. Internet: Diameter of the world-wide web. *Nature* **401**(6749), 130–131 (1999).

[7] Ebel, H., Mielsch, L. I., and Bornholdt, S. Scale-free topology of e-mail networks. *Phys. Rev. E* **66**, 035103(R) (2002).

[8] Leskovec, J., Lang, K. J., Dasgupta, A., and Mahoney, M. W. Community structure in large networks: Natural cluster sizes and the absence of large well-defined clusters. *Internet Math.* **6**(1), 29–123 (2009).

[9] Klimt, B. and Yang, Y. The enron corpus: A new dataset for email classification research. In *Machine Learning: ECML 2004*, 217–226. Springer (2004).

[10] https://www.cs.cmu.edu/~./enron/.

[11] Leskovec, J., Kleinberg, J., and Faloutsos, C. Graph evolution: Densification and shrinking diameters. *ACM Trans. Knowl. Discov. Data* **1**(1), 2 (2007).

[12] Gehrke, J., Ginsparg, P., and Kleinberg, J. Overview of the 2003 kdd cup. *ACM SIGKDD Explorations Newsletter* **5**(2), 149–151 (2003).

[13] http://vlado.fmf.uni-lj.si/pub/networks/data/.

[14] https://sites.google.com/site/andrealancichinetti/software.

[15] https://www-complexnetworks.lip6.fr/~latapy/PP/walktrap.html.

[16] http://igraph.org/python/.

[17] https://sites.google.com/site/findcommunities/.
